# In Vivo Neocortical [K]o Modulation by Targeted Stimulation of Astrocytes

**DOI:** 10.3390/ijms22168658

**Published:** 2021-08-12

**Authors:** Azin EbrahimAmini, Shanthini Mylvaganam, Paolo Bazzigaluppi, Mohamad Khazaei, Alexander Velumian, Bojana Stefanovic, Peter L. Carlen

**Affiliations:** 1 Krembil Research Institute, Toronto, ON M5T 0S8, Canada; shanthini.mylvaganam@uhnresearch.ca (S.M.); mkhazaei@uhnres.utoronto.ca (M.K.); carlen@uhnresearch.ca (P.L.C.); 2 Institute of Biomedical Engineering, University of Toronto, Toronto, ON M5S 3G9, Canada; 3 Sunnybrook Health Sciences Center, Medical Biophysics, Toronto, ON M4N 3M5, Canada; paolo.bazzigaluppi@sri.utoronto.ca (P.B.); bojana@sri.utoronto.ca (B.S.); 4 Division of Neurosurgery, University Health Network, Toronto, ON M5T 2S8, Canada; alexander.velumian@uhnresearch.ca; 5 Department of Surgery and Physiology, University of Toronto, Toronto, ON M5S 1A8, Canada; 6 Department of Medicine and Physiology, University of Toronto, Toronto, ON M5S 1A8, Canada

**Keywords:** astrocyte, extracellular potassium concentration, astrocyte membrane potential, halorhodopsin, hyperpolarization

## Abstract

A normally functioning nervous system requires normal extracellular potassium ion concentration ([K]o). Throughout the nervous system, several processes, including those of an astrocytic nature, are involved in [K]o regulation. In this study we investigated the effect of astrocytic photostimulation on [K]o. We hypothesized that in vivo photostimulation of eNpHR-expressing astrocytes leads to a decreased [K]o. Using optogenetic and electrophysiological techniques we showed that stimulation of eNpHR-expressing astrocytes resulted in a significantly decreased resting [K]o and evoked K responses. The amplitude of the concomitant spreading depolarization-like events also decreased. Our results imply that astrocytic membrane potential modification could be a potential tool for adjusting the [K]o.

## 1. Introduction

Neuronal activity leads to an elevation of extracellular potassium ion concentrations ([K]o). This must be regulated in order to prevent the adverse effects of abnormally high levels of [K]o, which include a wide range of neurological disorders [1]. Astrocytes play a critical role in [K]o regulation via several mechanisms such as astrocyte-mediated K buffering through astrocytic gap junctions and membrane proteins [2]. Among the membrane proteins involved in ionic regulation, the Kir4.1 channel is crucial for K buffering and for maintaining astrocytic membrane hyperpolarization [3]. Astrocytes also contribute to synaptic information processing through the release of gliotransmitters such as ATP and glutamate [1]. Glutamate as the main excitatory neurotransmitter, is required to be removed quickly from the extracellular space to prevent abnormal neuronal excitability. Glutamate transporters on astrocyte membranes are responsible for glutamate uptake from the extracellular environment, which is dependent on the hyperpolarization of astrocytic membrane potential [1]. Hyperpolarizing actuators such as archaerhodopsin-TP009 (ArchT) and halorhodopsin (NpHR) offer the possibility of studying the impact of astrocytic membrane hyperpolarization on enhancing the uptake of K and glutamate from the extracellular space. ArchT, which is a proton pump, allows for proton efflux upon photoactivation, leading to increased pH, alkalization, and astrocytic hyperpolarization. NpHR is a light-activated Cl^−^ pump allowing for Cl^−^ influx upon light illumination leading to membrane hyperpolarization [1]. Beppu et al. (2014) and Letellier et al. (2016) used ArchT to hyperpolarize astrocyte membranes in vitro with a focus on glutamate and gliotransmitters [4,5].

Beppu et al. (2014) recorded the astrocytic outward current and hyperpolarization from ArchT transgenic mice following glial ArchT photoactivation which led to a reduction in glutamate release, suppressing the hyperexcitation of nearby neurons and relieving ischemic brain damage. They suggested that the astrocytic alkalization and hyperpolarization resulting from ArchT photoactivation could be the cause of glutamate reduction. This phenomenon could also lead to K uptake from the extracellular space through Kir 4.1 or Kir 5.1 channels located on astrocyte membrane, therefore decreasing neuronal hyperexcitation [1]. Letellier et al. (2016) speculated that ArchT photoactivation alters synaptic transmission of presynaptic terminals via altered gliotransmission. They hyperpolarized the astrocytic membrane potential by photoactivation of ArchT-expressing hippocampal astrocytes in vitro. This resulted in astrocytic membrane hyperpolarization from about −78 to −102 mV [5].

In a study done by Tannesen et al. (2009), it was shown that NpHR photoactivation, hyperpolarized the transfected hippocampal principal neurons and suppressed in vitro epileptiform activity [6]. An in vivo optical-stimulation of neuronal and astrocytic channelrhodopsin (ChR)-expressing cells, which causes depolarization, resulted in transient elevation of [K]o by about 5 mM [7]. Significant increases in [K]o were maintained for the duration of the stimulation [7]. The authors suggested that astrocytic optical timulation increased neuronal excitability via [K]o elevation. Photostimulation of ChR-expressing cerebellar astrocytes triggered inward currents (depolarization) and the release of glutamate from the influx of protons in the ChR-expressing astrocytes [4,8].

Bentley et al. (2013) suggested that in theory it would be possible to control K uptake using hyperpolarizing rhodopsins [9]. In this study, we studied for the first time the effect of optical stimulation of eNpHR-expressing astrocytes on the in vivo resting [K]o and evoked K responses along with their concomitant local field potential (LFP) alterations. We show that the photostimulation of eNpHR expressing astrocytes under a short version of glial fibrillary acidic protein leads to decreased [K]o in vivo. 

## 2. Results

The PAAV-gfaABC1D-eNpHR3.1-eYFP genome was sequenced to confirm the existence of all segments; 156 to 836 label = gfaABC1D, 851 to 1681 label eNpHR3.1, 1682 to 2395 label eYFP. The full document is attached in the Supplementary Material. The Western blot gel showing the successful insertion of the plasmid is also attached in the Supplementary Material. 

**Figure 1:**
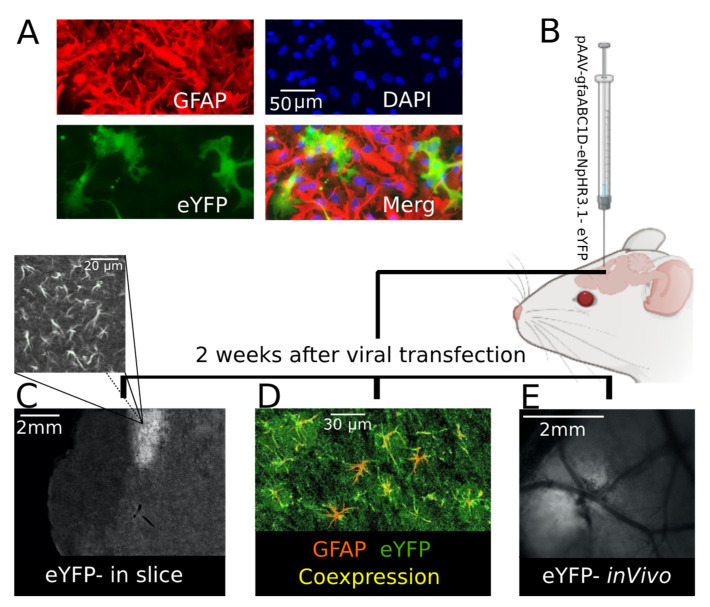
eYFP (conjugated with eNpHR) was confirmed to be expressed in mouse astrocytes. (**A**) Astrocyte-specific expression of eNpHR-eYFP (green) throughout the GFAP-positive astrocytes (red); they are shown as merged (yellow) to illustrate eNpHR and astrocyte colocalization in mouse cell culture. (**B**) CD-1 mouse viral transfection; 2 weeks after transfection. (**C**) Injection site expressing eYFP in a mouse brain slice; in the image above (**C**) it is magnified to show astrocytic-like processes. (**D**) Astrocytes (GFAP-positive (red)) expressing eNpHR (eYFP-conjugated (green)) shown in yellow to illustrate co-expression (yellow) in a mouse brain slice. (**E**) The injection site was distinguishable in vivo because of eYFP expression 2 weeks following mouse transfection.

Specific expression of eNpHR in the astrocytes was first tested in mouse cell culture. One week after viral transfection eNpHR-eYFP was reliably expressed in GFAP-positive cells, eNpHR was expressed in the astrocytes and cell nuclei stained positive for DAPI (Figure 1A). After cell culture, the expression of eNpHR-eYFP was studied in brain slice and in vivo. In this line, AAV carrying eNpHR-eYFP under the gfaABC promotor was microinjected into somatosensory cortex of 2 month-old CD-1 mice (Figure 1B). Two weeks later, eNpHR-eYFP was reliably expressed in the astrocytes of the transfected area (Figure 1C–E). After brain sectioning, eYFP was visualized in the injection site (Figure 1C), located in the area of astrocytic-like processes (Figure 1C above). For further confirmation using IHC, colocalization of GFAP and eYFP in brain slices was validated (Figure 1D). This observation confirmed that eNpHR was successfully expressed in the astrocytes, as eYFP was conjugated with eNpHR and astrocytes were GFAP-positive cells. EYFP expression allowed for distinguishing the virally transfected site in vivo (Figure 1E).

**Figure 2:**
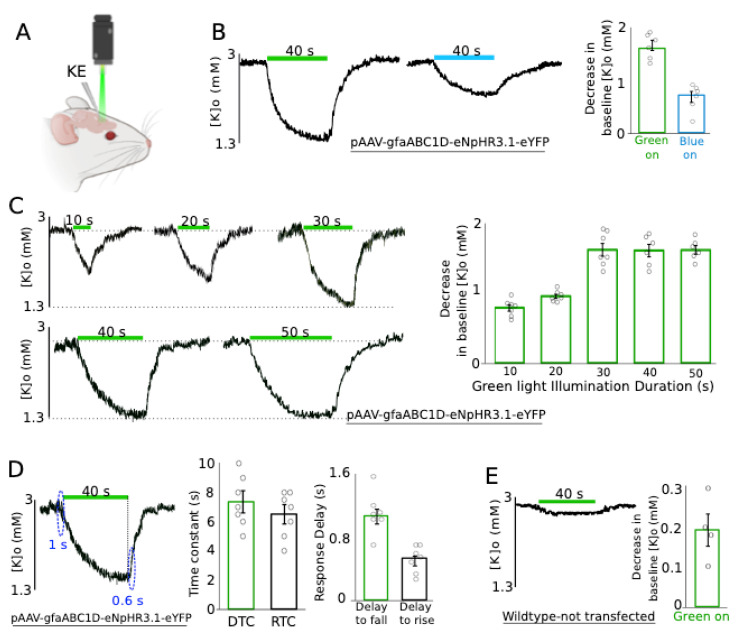
Green light application significantly decreased the resting [K]o in the transfected area. (**A**) An experimental configuration: 1 K-sensitive electrode (KE) was implanted in the transfected area to study the effect of green light application on [K]o. (**B**) Blue and green light with the same duration were tested on the transfected animal. Left: usage of blue light was not as effective as green light. Right: changes in the baseline [K]o amplitude following green and blue light application are summarized (*n =* 6). (**C**) Effect of green light application on [K]o with 5 different exposure times. Left: A gradual decrease in the resting [K]o; a minimum 30 s exposure time was required to see the [K]o decay reaching a plateau. Right: the reductions in the baseline [K]o exposed to different light durations are summarized (*n =* 7). (**D**) Middle: Decay time constant (DTC) and rise time constant (RTC) of the resting [K]o upon light application right: resting [K]o started falling/raising with a small delay (~1 s) after turning the light on/off (*n =* 7). (**E**) Control group: usage of green light on wildtype untransfected mice had a negligible effect on the baseline [K]o (*n =* 4). The green bar represents green light application.

To investigate the role of astrocytes on extracellular K redistribution, we measured the effect of the photostimulation of transfected astrocytes on the resting [K]o using a K-sensitive recording electrode (KE) in vivo within the transfected area (Figure 2A). To first establish the optimal eNpHR stimulation parameters we compared the effects of stimulation with two different wavelengths (green: 560 nm and blue: 460 nm), evidencing that resting [K]o decreased by 1.59 ± 0.09 mM and 0.71 ± 0.09 mM following green and blue light application, respectively (Figure 2B, *n* = 6), in the transfected animal. This confirmed that green light was the most effective for photostimulation of the transfected astrocytes; therefore, it was used for all further experiments. Next, we studied the impact of the light illumination duration on the previously observed [K]o decrease. For this purpose, green light illumination duration was categorized into five different groups—10, 20, 30, 40, and 50 s—resulting in a 0.79 ± 0.04, 0.95 ± 0.03, 1.61 ± 0.09, 1.60 ± 0.08, and 1.61 ± 0.06 mM decrease in the resting [K]o of the transfected animals (Figure 2C, *n =* 7), respectively. As shown, there was a gradual reduction in [K]o correlated with a light exposure duration up to 30 s, after which, the decrease in the [K]o reached a plateau and did not decrease further. Upon light illumination on the transfected area, the resting [K]o started falling with a decay time constant (DTC) of 7.4 ± 0.8, and started rising again with a rise time constant (RTC) of 6.5 ± 0.7 s (Figure 2D, left and middle, *n =* 7). The onset of the [K]o decrease (delay to fall) occurred 1.07 ± 0.09 s after light illumination, and started increasing (delay to rise) 0.53 ± 0.06 s after turning off the light source (Figure 2D, left & right, *n =* 7). To address the concern of heating damage and thermal effect on tissues exposed to light illumination, we illuminated green light on wildtype non-transfected CD-1 mice; consequently, [K]o decreased only by 0.19 ± 0.04 mM (Figure 2E, *n =* 4). Thus, the observed reduction in the resting [K]o is in fact due to eNpHR expression in the astrocytes.

**Figure 3:**
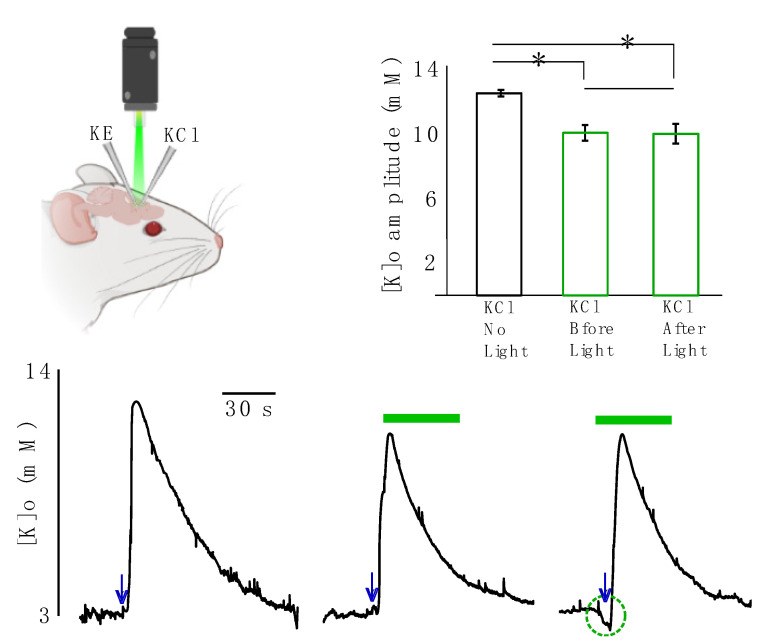
Effect of light application on focally evoked K responses (using KCl injection). KCl was focally applied either without any light, before light onset or after light onset. Top left: a K-sensitive recording electrode (KE) and a KCl injection micropipette (KCl) were implanted in the transfected area. Top right: changes in the amplitude of the evoked K response are summarized, black: in the absence of light. Green middle column: KCl was applied before light onset. Green right column: KCl was applied after light onset. * Represents significance (*p* < 0.05). Bottom: evoked K responses. Left: in the absence of light. Middle: KCl was applied before light onset. Right: KCl was applied after light onset. The green bar represents green light application, navy arrows represent time of KCl injection.

In the next step we studied the effect of light application on evoked K responses resembling pathological levels of [K]o. In these sets of experiments, the evoked K responses were generated by focal injection of 50 mM KCl solution and recorded by a single K-sensitive recording electrode (Figure 3, top right). The [K]o responses significantly decreased following light application by 2.5 ± 0.5 mM (from 12.6 ± 0.3 to 10.1 ± 0.5 mM, 20%, *p =* 0.01) if KCl was injected before light onset and by 2.6 ± 0.4 mM (from 12.6 ± 0.3 to 10.0 ± 0.5, 20.4% mM, *p =* 0.008) if KCl was injected after light onset (Figure 3, *n* = 6). This implies that the decreased amplitude of the evoked K response due to light application was independent of the start time of the light illumination, as the outcomes of the two scenarios were not significantly different from one another (*p =* 0.79). 

**Figure 4:**
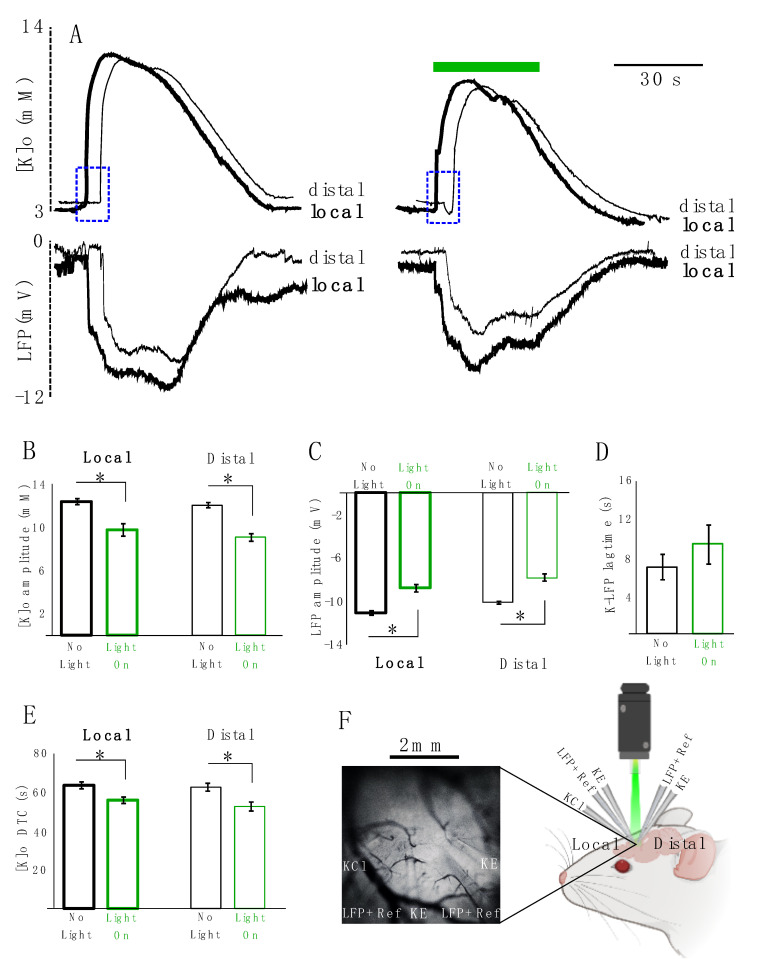
KCl-evoked K responses decreased in amplitude and DTC locally and distally. (**A**) Local and distal coupled K-LFP evoked responses: thick lines represent local responses (in the vicinity of the KCl injection site), thin lines represent distal response (~2 mm away from the injection site). Left: responses in the absence of light. Right: response in the presence of light. Top: [K]o responses. Bottom: LFP responses following KCl injection. (**B**) Summary of local and distal changes in the [K]o amplitude. (**C**) Summary of changes in the LFP amplitude locally and distally. (**D**) summary of changes in the lag time of the K-LFP spreading response traveling from the local to distal site indicated in dotted blue rectangles. (**E**) Summary of changes in the local and distal DTC. Black is in the absence of light and green is when the transfected tissue is exposed to green light illumination. “*’’ represents significance (*p* < 0.05). (**F**) Right: experimental configuration: 2 sets of coupled KE-LFP recording electrodes were placed about 2 mm apart from one another, and a KCl injection electrode (KCl) was implanted in the vicinity of one of the KE-LFP electrodes (local site). K-sensitive recording electrode (KE). Local field potential recording electrode along with a local reference electrode for KE (LFP + Ref). Left: in vivo electrodes setting implanted in the transfected area expressing eYFP (*n =* 5).

Next, we studied the effect of astrocytic photo-stimulation on [K]o redistribution and the concomitant local field potential (LFP) responses (*n =* 5). For this purpose, K-sensitive recording electrodes (KE) were coupled with LFP recording electrodes along with local references for K electrodes. Two sets of these coupled electrodes were placed approximately 2 mm apart from each other and a KCl microinjection pipette was placed in the vicinity of one of the two. The recording site closer to the KCl microinjection pipette was considered as the local site and the one 2 mm apart from it was considered as the distal site (Figure 4F). We measured the amplitude, decay time constant (DTC) and lag time (the time that it takes the evoked response to travel from the local to distal site) of the spreading evoked K responses coupled with their concomitant LFP responses. Following optical stimulation of transfected astrocytes, the amplitude of the K response decreased significantly locally by 2.6 ± 0.4 mM (from 12.5 ± 0.3 to 9.8 ± 0.6 mM, 21%, *p =* 0.021) and distally by 3.0 ± 0.2 mM (from 12.1 ± 0.2 to 9.1 ± 0.3 mM, 25%, *p* = 0.012) following light application (Figure 4A top and B). Concurrently, the amplitude of the LFP response also decreased significantly by 2.4 ± 0.3 mV (from 11.2 ± 0.2 to 8.9 ± 0.3 mV, 21%, *p =* 0.012) locally and by 2.2 ± 0.3 mV (from 10.2 ± 0.1 to 8 ± 0.3 mV, 22%, *p =* 0.012) distally (Figure 4A bottom, C). However, the lag time of the spreading KE-LFP response did not significantly increase after light application (by 2.4 ± 0.8 s, from 6.8 ± 1.3 to 9.2 ± 1.9, 26%; *p =* 0.4) (Figure 4A: blue rectangles and D). The DTC of the K response significantly decreased by 7.7 ± 2.3 s (from 63.5 ± 1.8 to 55.8 ± 1.7 s, 12%, *p =* 0.028) locally and by 10.0 ± 2.4 s (from 62.5 ± 2 to 52.6 ± 2.3 s, 16%, *p =* 0.021) distally (Figure 4A,E). DTC changes of the concomitant LFP responses were not significant. 

**Figure 5:**
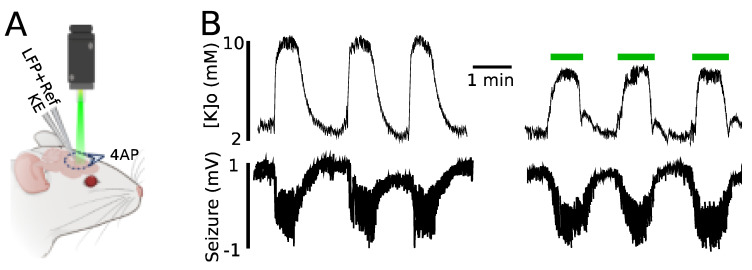
Evoked K responses triggered by 4AP reduced in amplitude following green light application. (**A**) Experimental configuration: topical 4AP application, K recording electrode (KE), local field potential recording electrode along with a local reference electrode for KE (LFP + Ref). (**B**) Top: [K]o during seizure activity in the presence and absence of light. Bottom: seizures induced by topical 4AP application recorded by LFP.

We further investigated the impact of astrocytic optical stimulation on 4AP-evoked K responses as an example of a pathophysiological elevation of [K]o. A K-sensitive recording electrode (KE) possessing a local reference and coupled with a LFP electrode allowed for simultaneous recording of [K]o and seizure activity (Figure 5A). In line with previous findings, light application led to a significant reduction in the amplitude of evoked K responses (by 2.2 ± 0.1 mM, from 9.9 ± 0.4 to 7.8 ± 0.5 mM, *n =* 3) as compared to the no-light scenario (Figure 5B). The effect of light on 4AP-evoked seizure activity was not significant (amplitude from 1.8 ± 0.1 to 1.7 ± 0.15 mV; duration from 56 ± 6 to 58 ± 4 s). 

## 3. Discussion

Abnormally high levels of [K]o are associated with neurological conditions such as neurotrauma, migraine, stroke, and epilepsy [10,11]. Astrocytes are known to be one of the major factors in regulating [K]o. Astrocytes have a hyperpolarized resting membrane potential and a high permeability to K [12]. In this study, we investigated the impact of optical activation of eNpHR expressing astrocytic on [K]o. The results of this study imply that the astrocytic membrane potential has a significant role in [K]o dynamics. 

We showed that optical stimulation of eNpHR-expressing astrocytes resulted in a significant decrease in the amplitude of the resting and evoked K responses triggered by KCl focal injection or 4AP topical application. The DTC of the evoked K responses also decreased significantly following light application. The lag time increase was not significant. The evoked K responses were partially suppressed after light application by a few mM (~2.5 mM), somewhat more than the light-evoked decrease from resting [K]o (~1.6 mM). This observation probably indicates the existence of auxiliary mechanisms which were activated only by abnormally high levels of [K]o. The decrease in the amplitude of the distal evoked K responses (~3 mM and 10 s) was more prominent than the decreases in the local responses (2.6 mM and 7.7 s), likely because the already-suppressed response was reaching the distal site. Application of light on the non-transfected astrocytes in control conditions did not have a significant effect on the [K]o; the effect was only evident when astrocytes were expressing eNpHR. Thus, the decrease in [K]o was due to optical stimulation of transfected astrocytes. 

In the presence of light, resting [K]o with a DTC of 7.4 ± 0.8 s and RTC of 6.6 ± 0.7 s decreased gradually up until about 30 s of light exposure, after which, the decrease in the [K]o reached a plateau and did not decrease further. This implies that: (1) astrocytes have a limited capacity for re-uptake of K from the extracellular space, so further photo-stimulation would be ineffective and/or the process activated by the photostimulation is quite slow; (2) the existing K in the extracellular space is only partially regulated/taken up by astrocyte membrane potential, and therefore other regulatory mechanism are involved; and/or (3) longer photo-stimulation does not translate to more hyperpolarization as cells cannot be hyperpolarized beyond a certain voltage. 

The concomitant LFP responses coupled with the evoked K responses were negative DC shifts representing spreading depolarizations (SDs) (Figure 4). These SD-like events decreased in amplitude following optical stimulation. Failing to observe a total suppression of SD-like events could be explained by the partial and incomplete decrease in the [K]o and emphasizes the involvement of other processes in shaping SDs. 

Despite the significant decrease in the 4AP-induced K responses caused by light application, concomitant seizures were not significantly affected (Figure 5). Not seeing a significant change in the seizure activity following light application could be due to multiple factors; first and foremost probably the small local decrease (~2 mM) of the already raised [K]o was not influential enough to modify the robust seizure activities being generated from a much larger area of topically-applied 4AP, i.e., seizures were almost certainly triggered from multiple surrounding sites, and therefore illuminating light on a small area would not be able to modify the activity.

The decay time and amplitude of the K response were affected more than the lag time of the spreading responses following optical stimulation. This observation could imply that the astrocytic membrane potential presumably plays a more significant role in regulating the resident/local [K]o and adjusting the concentration of the existing K in the local extracellular space rather than the global distribution of K, whereas astrocytic gap junctional coupling has a more prominent role in speeding up the spread of the spreading K response [11].

As studied by Djukic et al. (2007) Ba^+^-mediated blockade and conditional RNAi-mediated knockdown of Kir4.1 hinder K uptake from the extracellular space, depolarize the astrocytic membrane, and lead to seizures [13]. In Kir4.1 knockout mice the astrocyte membrane potential is severely affected, being reduced from 85 to 13 mV [14]. Tong et al. (2014) showed that Kir4.1 restoration rescues channel conductance and membrane potential in a Huntington’s disease mice model which exhibited depolarized membrane potential and reduced K buffering caused by decreased Kir4.1 functional expression [15]. 

Therefore, according to our study and the reviewed literature, we propose that our observation of decreased [K]o is due to Kir4.1 activation following astrocytic membrane hyperpolarization caused by photostimulation [12,16,17,18,19,20,21]. Following illumination with green light, eNpHR as a chloride pump causes chloride ions to enter the cell, leading to cell membrane hyperpolarization [1,22]. This change in the astrocyte’s membrane potential activates astrocytic membrane proteins such as K inwardly rectifying (Kir) channels [16,17,18], and leads to K reuptake from the extracellular space. Although we did show that eNpHR was expressed in the astrocytes and we already know that eNpHR causes membrane hyperpolarization [23], measuring astrocytic hyperpolarization in the in vivo experiments was not feasible in this study. 

One could also argue that green light illumination on eNpHR-expressing astrocytes, which leads to increased intracellular chloride concentrations, is a driving force for the influx of positive current (i.e., K), therefore decreasing [K]o. This implies the possibility that hyperpolarization itself caused by eNpHR photoactivation (chloride influx) or the very small amount of K influx via eNpHR openings could be the actual trigger for decreasing [K]o. 

Extracellular K and glutamate concentration are related to one another, as transporting glutamate into the cell is accompanied by the extrusion of K [24]. K release is known to be compulsory for starting a new glutamate transport cycle and abnormally high levels of [K]o have an immediate inhibitory effect on glutamate transporters [24]. Meeks and Mennerick (2004) suggested that glutamatergic transmission in the CA1 was significantly depressed by 8–10 mM of [K]o but GABAergic transmission remained unchanged [25]. Glutamate transporters on the astrocyte membrane are responsible for glutamate uptake from the extracellular environment which is dependent on the hyperpolarization of the astrocytic membrane potential [1]. Thus, astrocytic hyperpolarization resulting from eNpHR photoactivation could also lead to extracellular glutamate reduction in addition to K uptake from the extracellular space through Kir channels thereby decreasing neuronal hyperexcitation.

## 4. Methods

### 4.1. Viral Construct

An adeno-associated virus (AAV) vector carrying an enhanced Halorhodopsin *3.1* (eNpHR) gene was tagged with enhanced yellow fluorescent protein (eYFP) as a gene reporter and was conjugated with an astrocytic specific gene promoter known as gfaABC1D which is a short version of glial fibrillary acidic protein (GFAP or GFAP104), at the the Goshen laboratory. The plasmids were amplified and purified in the Carlen laboratory at the Krembil Research Institute. Plasmids were then sent to the University of Pennsylvania for insertion into the viral construct. 

### 4.2. Plasmid Amplification

The One Shot™ Stbl3™ Chemically Competent E. coli (Catalog Number C7373-03) kit was used to transform the plasmid. Most of the steps were followed according to the company manual but the antibiotic and the incubation temperatures were modified. LB medium and LB agar plates were developed with 100 μg/mL carbenicillin. Plasmid DNA, (approximatly 1–50 ng) was used for each transformation and PUC19 (provided in the kit) was used as a control. After the DNA was transformed it was plated on an LB agar + carbenicillin plate and incubated at 30 °C overnight. Single colonies were picked from the plates and grew in several 500 mL LB + carbenicillin media plates overnight. The cells were spun down and large-scale plasmid preps created using the QIAGEN plasmid Maxi Kit (cat. no. 12162). The protocol was followed according to the manual. Then each prep was quantified and the presence of the inserts was confirmed by digesting them with restriction enzymes Not1 and Sma. The sizes from the restriction—NotI: 2887, 1902, and 1531 bp, SmaI: 2937, 2681, 680, 11bp—were matched with the expected sizes. Finally, all the plasmid DNAs were pooled and sent to Penn State for production of the adenovirus.

### 4.3. Animals

Experiments were conducted on 1–2 month-old, 18–30 g CD-1 mice. Animals were housed in a 12/12 h light cycle with ad libitum access to water and food. All experimental procedures performed in vivo were approved by the Krembil Research Institute.

### 4.4. Viral Transfection

Two weeks prior to the experiments, the pAAV-gfaABC1D-eNpHR3.1-EYFP construct was stereotactically injected into the mouse cortex. The genome titer of the concentrated virus for in vivo injection was 3.072 × 10^13^ GC/m with a 2.55 × 10^13^ GC yield. Aseptic procedures were followed throughout the surgery. CD-1 mice were anesthetized with isoflurane. Once induced, they were placed into a stereotactic frame and an ophthalmic ointment applied to the eyes. The mouse’s skull was shaved, wiped with betadine, and rinsed with 70% alcohol. Subcutaneous baytril (10 mg/kg) was given subcutaneously. An incision was made in the scalp, and the underlying fascia retracted. Using a micro-drill, a burr hole was drilled in the skull over the hind limb somatosensory cortex (intracerebroventricular coordination: anterior-posterior: −0.5 mm, medial-lateral: 1.5 mm, dorsal-ventral: 2.2 mm). Then, 1.5 μL of virus was injected at 0.15 μL/min into the cortex using a Hamilton syringe and a stereotaxic syringe holder. After the injection was completed, there was a waiting period of 3 min while the syringe was inside the brain to avoid any backsplash and ensure complete absorption. Upon completion of surgery, sterile bone wax was applied to the skull, the scalp was sutured, intraperitoneal dexamethasone (1 mg/kg) was administered, and isoflurane discontinued. Mice recovered under a warming lamp and were given saline every 4 h as needed. They were checked for bleeding, abnormal behavior, or signs of infection/inflammation during this period. 

### 4.5. Craniotomy

Two weeks after cortical viral transfection, a craniotomy was performed with a precision drill, removing a circular region of the skull measuring 5 mm in diameter over the right somatosensory cortex (transfection site).Mice were anesthetized with 5% inhaled isoflurane with oxygen flowing at 1 mL/min to induce anesthesia. During the surgery inhaled isoflurane was reduced to 1.5–2% and oxygen to 0.5 mL/min. Phosphate buffered saline (PBS) was applied over the exposed cortex, filling the cavity of the skull, to prevent tissue damage and dehydration. The animal body temperature was maintained at 37.5 °C using a heating pad Hind limb withdrawal reflexes and breathing rate were observed at regular intervals throughout the experiment to ensure that the animal remained at a surgical plane of anesthesia. After craniotomy, the mice were transferred to the recording chamber, which was a darkened enclosure.

### 4.6. Electrophysiology: K-Sensitive Recording Electrode

In order to maintain accurate measurements of K by the K-sensitive electrodes (KE), it is necessary to account for the component arising from an electrical field. To this end, a local reference electrode was used to mitigate distortion of K-sensitive electrode readings. The electrodes were pulled borosilicate capillaries (tip diameter ~1 μm, World Precision Instruments, Sarasota, FL, USA). In the case of the K-sensitive electrode, the interior wall of the capillary was silanized with dimethyldichlorosilane vapor and dried at 120 °C for 2 h [11]. K-sensitive electrodes were filled with K^+^ ionophore I-cocktail B (Sigma-Aldrich, Oakville, ON, Canada) at the tip and back-filled with 0.2 M KCl solution [11]. The K-sensitive recording electrodes were then calibrated using different concentrations (0, 2.5, 4.5, 6.5 and 22.5 mM) of KCl solutions. The relationship between the measured voltage and the K concentration of the respective solution was derived using the Nicolsky—Eisenmann equation [26] which is a commonly used method. The latter calibration lines which were semi-logarithmic and close to linear, were used to determine [K]o in the brain [27].

### 4.7. Electrophysiology: 2(KE-LFP) Electrode and KCl

A K-sensitive electrode (KE) was coupled with a double-barreled LFP recording electrode, creating a KE-LFP recording electrode. The double-barreled electrode was filled with saline and cemented to the K-sensitive electrode such that the distance between the tips of the electrodes was approximately 50 μm apart [11]. First, the K-sensitive electrode was mounted to a head stage of an Axopatch 200B amplifier. A differential reference electrode for the head stage was inserted into a chamber of the double-barreled LFP electrode; the other chamber was used to record the extracellular LFP connected to a head-stage of an Axopatch 200B amplifier. This latter signal was differentially recorded from a common ground wire, attached to the scalp. This arrangement was duplicated to have a KE-LFP recording available at each recording site. All amplifiers were then digitized (Digidata 1440, Molecular Devices). LFP and extracellular K signals were low-pass filtered at 5 kHz. This arrangement allowed for simultaneous and effective recordings of LFP and evoked [K]o responses (KE-LFP response) from the same location in the cortex caused by KCl injections. Electrodes were lowered into the cortex in steps of 0.1 mm. Under an Olympus BX-61W1 microscope with 4X PlanN objectives, 2 sets of KE-LFP electrodes, were inserted into the right somatosensory cortex (site of viral transfection- where eYFP was expressed), such that their tips were approximately 2 mm apart in a horizontal plane. An injection micropipette filled with 50 mM KCl, was placed near one of the KE-LFP recording electrodes. After 10 min of recording at baseline, the focal application of the KCl was performed by 3 repetitions (every 0.3 s) of 5–7 ms microinjections (PicoSpritzer III, Parker), resulting in a total injection volume of about 1 μL. The recording site immediately adjacent to the KCl injection site is referred to as the local site, whereas the other recording site is referred to as the distal site.

### 4.8. KE-LFP Electrode and 4AP

This time only one of the coupled KE-LFP was used to record the evoked K responses and the seizure activity, which was caused by topical application of 200 μL of 5 mM 4-aminopyridine (4AP) solution [28]. 

### 4.9. Optogenetics (eNpHR3.1 Activation)

Enhanced Halorhodopsin 3.1 (eNpHR) is a light-activated chloride ion pump [6] with peak absorption at 570 nm (wavelength between 560 and 650 nm) [29]. Light with a 570 nm wavelength was illuminated on the transfected area by passing from a 200 W mercury fluorescent lamp via standard microscope objectives. Light application was controlled via an electrical shutter.

### 4.10. Mouse Astrocyte Culture

Newborn (P5) mice were decapitated. The cortex hemispheres were isolated in ice-cold PBS and the meninges were removed. The tissues were incubated with TrypLE Express enzyme for 10 min at 37 °C. The cortices were then triturated by pipetting up and down using fire-polished Pasteur pipettes. The cell suspension was filtered through a 40 μm cell strainer. Cells were re-suspended in astrocyte culture medium (DMEM, high glucose + 10% heat-inactivated fetal bovine serum + 1% penicillin/streptomycin), plated in T75 flask pre-coated poly-D-lysine (PDL) (20 mL at a concentration of 50 μg/mL) and cultured at 37 °C and 5% CO_2_. The culture media was changed every 3 days for 9 days. In the next step, overlaying microglia that were on top of the astrocyte layer were detached by shaking the T75 flask at 180 rpm for 30 min on an orbital shaker. The media was changed to 20 mL fresh astrocyte culture medium and flasks were shaken at 240 rpm for 6 h to remove oligodendrocyte precursor cells (OPC). Next step, the media was changed to fresh media and flasks were shaken vigorously by hand for 1 min in order to remove any potential OPC contamination. The remaining confluent astrocyte layer were rinsed twice with PBS, and then dissociated with TrypLE Express enzyme for 5 min at 37 °C. TrypLE was neutralized with astrocyte culture medium and then the cells were plated at a density of 4 × 10^4^ cells/mL on PDL-coated plates. 

### 4.11. Immunohistochemistry

Two weeks after viral transfection, animals were anesthetized using a 100 μL mixture of avertin and ketamine. Then mice were transcardially perfused with PBS followed by 4% paraformaldehyde (PFA). Mouse brains were collected and stored in 4% PFA overnight at 4 °C on a shaker. The next day brains were placed in PBS 3 times each for 30 min then transferred in 30% sucrose overnight. Brains were cut to 20 μm sections using a cryostat. Then, 300–500 μL of 0.1% Triton was applied on the slides twice. Subsequently 500 μL of Triton and 5% bovine serum albumin (BSA) were added to the slides. Primary antibody (mice anti-GFAP, 1:500) in Triton and 1% BSA was applied to the slides and kept overnight at 4 °C. It was then washed twice with PBS. Secondary antibody (anti-mice Alexa flour488, 1:200) diluted in Triton and 1% BSA was applied and kept for 2 h. This was washed 3 times with PBS. DAPI was added to the slides and left for 10 min, and rinsed once with PBS. Then, images were captured on a confocal microscope (BX61W1, Olympus). 

### 4.12. Statistical Analysis

Data analysis was performed using MATLAB 2016. Considering the low number of data samples, non-parametric statistical analysis was performed. A two-tailed hypothesis was tested, and the p-value was calculated using the Wilcoxon-Mann-Whitney test. A *p*-value below 0.05 was considered significant. Statistical analysis was performed to evaluate changes in amplitude, decay time constant (DTC) and lag time (time difference between the onset of the local and distal responses) for each animal. Results were reported as group average ± SEM; percent changes was also reported. 

## 5. Conclusions

In conclusion, according to our observations and the reviewed literature, we propose that our observations of decreased resting and evoked [K]o were almost certainly due to influx of K into astrocytes caused by photo-stimulation of eNpHR-expressing astrocytes. The presumed mechanism is that astrocytic membrane hyperpolarization increases the driving force for intake of K from the extracellular space. Yet, the exact role of astrocytic membrane potential in [K]o regulation needs further investigation. This study highlights the role of the astrocytic membrane potential in K regulation. This study suggests optogenetic astrocytic modulation as a tool for controlling [K]o and for developing strategies to understand and treat K-related neurological disorders.

## Data Availability

The data presented in this study are available on request from the corresponding author.

## Supplementary Materials

The following are available online at https://www.mdpi.com/article/10.3390/ijms22168658/s1.
