# Supplementary material for: In Vivo Neocortical [K]o Modulation by Targeted Stimulation of Astrocytes
Source: Int J Mol Sci. 2021 Aug 12;22(16):8658. doi: 10.3390/ijms22168658 (PMC8395460; doi:10.3390/ijms22168658)
Supplement: Supplementary file 1 [file ijms-22-08658-s001.zip › ijms-1320331-supplementary.pdf]

Supplementary

```
LOCUS       pAAV-gfaABC1D-eNpHR3.1-eYFP.xdna    6320 bp     DNA     circular   9/20/2016
DEFINITION
ACCESSION
VERSION
SOURCE
  ORGANISM
COMMENT     Serial Cloner Genbank Format
COMMENT     SerialCloner_Type=DNA
COMMENT     SerialCloner_Comments=Ligation of : pAAV-gfABC1D-EYFP (2).xdna [4730  nt] : (EcoRI
          [1569] / AgeI[837]) to pLenti-CaMKIIa-eNpHR_3.xdna [1693  nt
          ] : (AgeI[3906] / EcoRI[5603])
COMMENT     SerialCloner_Ends=0,0,,0,
FEATURES             Location/Qualifiers
     misc_feature    156..836
                     /label=gfaABC1D
                     /SerialCloner_Color=&hFF8040
                     /SerialCloner_Show=True
                     /SerialCloner_Protect=True
                     /SerialCloner_Arrow=True
     misc_feature    2437..3045
                     /label=WPRE
                     /SerialCloner_Color=&hFF0080
                     /SerialCloner_Show=True
                     /SerialCloner_Protect=True
                     /SerialCloner_Arrow=True
     misc_feature    1682..2395
                     /label=eYFP
                     /SerialCloner_Color=&h80FF00
                     /SerialCloner_Show=True
                     /SerialCloner_Protect=True
                     /SerialCloner_Arrow=True
     misc_feature    851..1681
                     /label=eNpHR3.1
                     /SerialCloner_Color=&h0080FF
                     /SerialCloner_Show=True
                     /SerialCloner_Protect=True
                     /SerialCloner_Arrow=True
     rep_origin      5649..6277
                     /label=ColE1 origin
                     /SerialCloner_Color=&h4682B4
                     /SerialCloner_Show=True
```

```
                     /SerialCloner_Protect=False
                     /SerialCloner_Arrow=True
rep_origin      complement(3803..4243)
                     /label=F1 ori
                     /SerialCloner_Color=&h4682B4
                     /SerialCloner_Show=True
                     /SerialCloner_Protect=False
                     /SerialCloner_Arrow=True
rep_origin      3798..4253
                     /label=M13 origin
                     /SerialCloner_Color=&h4682B4
                     /SerialCloner_Show=True
                     /SerialCloner_Protect=False
                     /SerialCloner_Arrow=True
CDS             4838..5497
                     /label=AmpR
                     /SerialCloner_Color=&h8FBC8F
                     /SerialCloner_Show=True
                     /SerialCloner_Protect=False
                     /SerialCloner_Arrow=True
polyA_signal    3066..3546
                     /label=hGH polyA signal
                     /SerialCloner_Color=&hFFC0CB
                     /SerialCloner_Show=True
                     /SerialCloner_Protect=False
                     /SerialCloner_Arrow=True
Promoter        4570..4598
                     /label=Amp prom
                     /SerialCloner_Color=&h4682B4
                     /SerialCloner_Show=True
                     /SerialCloner_Protect=False
                     /SerialCloner_Arrow=True
misc_feature    1..141
                     /label=L-ITR
                     /SerialCloner_Color=&hC0C0C0
                     /SerialCloner_Show=True
                     /SerialCloner_Protect=True
                     /SerialCloner_Arrow=True
misc_feature    complement(3583..3723)
                     /label=R-ITR
                     /SerialCloner_Color=&hC0C0C0
                     /SerialCloner_Show=True
                     /SerialCloner_Protect=True
                     /SerialCloner_Arrow=True
```

```
primer_bind    577..597
             /label=sgfap_midF
             /SerialCloner_Color=&h808080
             /SerialCloner_Show=True
             /SerialCloner_Protect=True
             /SerialCloner_Arrow=True
primer_bind    1724..1745
             /label=YFP_R
             /SerialCloner_Color=&hC0C0C0
             /SerialCloner_Show=True
             /SerialCloner_Protect=True
             /SerialCloner_Arrow=True
primer_bind    2500..2520
             /label=WPRE_R
             /SerialCloner_Color=&hC0C0C0
             /SerialCloner_Show=True
             /SerialCloner_Protect=True
             /SerialCloner_Arrow=True
primer_bind    2214..2235
             /label=eYFP_3_F
             /SerialCloner_Color=&hC0C0C0
             /SerialCloner_Show=True
             /SerialCloner_Protect=True
             /SerialCloner_Arrow=True
misc_feature   3127..3149
             /label=polyA_5_F
             /SerialCloner_Color=&hC0C0C0
             /SerialCloner_Show=True
             /SerialCloner_Protect=True
             /SerialCloner_Arrow=True
primer_bind    3477..3500
             /label=polyA_3_R
             /SerialCloner_Color=&hC0C0C0
             /SerialCloner_Show=True
             /SerialCloner_Protect=True
             /SerialCloner_Arrow=True
primer_bind    1213..1234
             /label=eNpHR_F
             /SerialCloner_Color=&hC0C0C0
             /SerialCloner_Show=True
             /SerialCloner_Protect=True
             /SerialCloner_Arrow=True
misc_feature   2396..2419
             /label=Export signal + Stop
```

```
                 /SerialCloner_Color=&hFF00FF
                 /SerialCloner_Show=True
                 /SerialCloner_Protect=True
                 /SerialCloner_Arrow=True
                 /SerialCloner_Desc=note="direct strand translation"translation="FCYENEV" \
ORIGIN
        1 cctgcaggca gctgcgcgct cgctcgctca ctgaggccgc ccgggcaaag cccgggcgtc
       61 gggcgacctt tggtcgcccg gcctcagtga gcgagcgagc gcgcagagag ggagtggcca
      121 actccatcac taggggttcc tgcggccgca cgcgtaacat atcctggtgt ggagtagggg
      181 acgctgctct gacagaggct cgggggcctg agctggctct gtgagctggg gaggaggcag
      241 acagccaggc cttgtctgca agcagacctg gcagcattgg gctggccgcc ccccagggcc
      301 tcctcttcat gcccagtgaa tgactcacct tggcacagac acaatgttcg gggtgggcac
      361 agtgcctgct tcccgccgca ccccagcccc cctcaaatgc cttccgagaa gcccattgag
      421 caggggggctt gcattgcacc ccagcctgac agcctggcat cttgggataa aagcagcaca
      481 gccccctagg ggctgccctt gctgtgtggc gccaccggcg gtggagaaca aggctctatt
      541 cagcctgtgc ccaggaaagg ggatcagggg atgcccaggc atggacagtg ggtggcaggg
      601 ggggagagga gggctgtctg cttcccagaa gtccaaggac acaaatgggt gaggggagag
      661 ctctccccat agctgggctg cggcccaacc ccacccccctc aggctatgcc agggggtgtt
      721 gccaggggca cccgggcatc gccagtctag cccactcctt cataaagccc tcgcatccca
      781 ggagcgagca gagccagagc aggttggaga ggagacgcat cacctccgct gctcgcaccg
      841 gtaggccacc atggttaccc aaagggagtt gttcgagttc gtgctgaacg accctttgct
      901 tgcaagcagt ctctatatca acatcgcact tgcaggactg agtatactgc tgttcgtttt
      961 tatgacccga ggactcgatg atccacgggc aaaacttatt gctgtgtcaa ccatccttgt
     1021 gcctgtcgtc agcattgcct cctacactgg attggcgagc ggcctgacaa tttccgttct
     1081 tgaaatgcca gcgggccatt ttgcagaagg cagctcagtg atgctgggag gagaagaggt
     1141 agatggtgta gtcaccatgt ggggacggta tctcacctgg gcactttcca cgcccatgat
     1201 tctcctcgct ctgggtctcc tggccggaag caatgctaca aagctcttca cagctatcac
     1261 tttcgatatc gctatgtgcg tgactggcct tgccgcggcc ctgactacct cctcccacct
     1321 catgagatgg ttctggtacg ctatcagttg tgcatgcttt ctggtggtct tgtatatcct
     1381 gctggtggag tgggcacagg acgccaaagc cgcgggaacc gctgacatgt tcaataccct
     1441 gaagctgttg acagtagtga tgtggctggg gtatccaatt gtgtgggctc ttggagtcga
     1501 gggtatcgcg gtgttgcccg ttggggtgac gagctgggga tattctttcc tggatatcgt
     1561 ggcaaagtac attttcgcat tcttgctcct gaactatctg acgtcaaacg aatctgtcgt
     1621 gtccggcagc attttggatg ttccatctgc ttctgggacc ccggctgatg atgcggccgc
     1681 cgtgagcaag ggcgaggagc tgttcaccgg ggtggtgccc atcctggtcg agctggacgg
     1741 cgacgtaaac ggccacaagt tcagcgtgtc cggcgagggc gagggcgatg ccacctacgg
     1801 caagctgacc ctgaagttca tctgcaccac cggcaagctg cccgtgccct ggcccaccct
     1861 cgtgaccacc ttcggctacg gcctgcagtg cttcgcccgc taccccgacc acatgaagca
     1921 gcacgacttc ttcaagtccg ccatgcccga aggctacgtc caggagcgca ccatcttctt
     1981 caaggacgac ggcaactaca agacccgcgc cgaggtgaag ttcgagggcg acaccctggt
     2041 gaaccgcatc gagctgaagg gcatcgactt caaggaggac ggcaacatcc tggggcacaa
     2101 gctggagtac aactacaaca gccacaacgt ctatatcatg gccgacaagc agaagaacgg
     2161 catcaaggtg aacttcaaga tccgccacaa catcgaggac ggcagcgtgc agctcgccga
     2221 ccactaccag cagaacaccc ccatcggcga cggccccgtg ctgctgcccg acaaccacta
```

2281 cctgagctac cagtccgccc tgagcaaaga ccccaacgag aagcgcgatc acatggtcct

2341 gctggagttc gtgaccgccg ccgggatcac tctcggcatg gacgagctgt acaagttctg

2401 ctacgagaac gaggtgtaat gagaattcga tatcaagctt atcgataatc aacctctgga

2461 ttacaaaatt tgtgaaagat tgactggtat tcttaactat gttgctcctt ttacgctatg

2521 tggatacgct gctttaatgc ctttgtatca tgctattgct tcccgtatgg ctttcatttt

2581 ctcctccttg tataaatcct ggttgctgtc tctttatgag gagttgtggc ccgttgtcag

2641 gcaacgtggc gtggtgtgca ctgtgtttgc tgacgcaacc cccactggtt ggggcattgc

2701 caccacctgt cagctccttt ccgggacttt cgctttcccc ctccctattg ccacggcgga

2761 actcatcgcc gcctgccttg cccgctgctg gacaggggct cggctgttgg gcactgacaa

2821 ttccgtggtg ttgtcgggga aatcatcgtc ctttccttgg ctgctcgcct gtgttgccac

2881 ctggattctg cgcgggacgt ccttctgcta cgtcccttcg gccctcaatc cagcggacct

2941 tccttcccgc ggcctgctgc cggctctgcg gcctcttccg cgtcttcgcc ttcgccctca

3001 gacgagtcgg atctcccttt gggccgcctc cccgcatcga taccgagcgc tgctcgagag

3061 atctacgggt ggcatccctg tgacccctcc ccagtgcctc tcctggccct ggaagttgcc

3121 actccagtgc ccaccagcct tgtcctaata aaattaagtt gcatcatttt gtctgactag

3181 gtgtccttct ataatattat ggggtggagg ggggtggtat ggagcaaggg gcaagttggg

3241 aagacaacct gtagggcctg cggggtctat tgggaaccaa gctggagtgc agtggcacaa

3301 tcttggctca ctgcaatctc cgcctcctgg gttcaagcga ttctcctgcc tcagcctccc

3361 gagttgttgg gattccaggc atgcatgacc aggctcagct aatttttgtt tttttggtag

3421 agacggggtt tcaccatatt ggccaggctg gtctccaact cctaatctca ggtgatctac

3481 ccaccttggc ctcccaaatt gctgggatta caggcgtgaa ccactgctcc cttccctgtc

3541 cttctgattt tgtaggtaac cacgtgcgga ccgagcggcc gcaggaaccc ctagtgatgg

3601 agttggccac tccctctctg cgcgctcgct cgctcactga ggccgggcga ccaaaggtcg

3661 cccgacgccc gggctttgcc cgggcggcct cagtgagcga gcgagcgcgc agctgcctgc

3721 aggggcgcct gatgcggtat tttctcctta cgcatctgtg cggtatttca caccgcatac

3781 gtcaaagcaa ccatagtacg cgccctgtag cggcgcatta agcgcggcgg gtgtggtggt

3841 tacgcgcagc gtgaccgcta cacttgccag cgccctagcg cccgctcctt tcgctttctt

3901 cccttccttt ctcgccacgt tcgccggctt tccccgtcaa gctctaaatc gggggctccc

3961 tttagggttc cgatttagtg ctttacggca cctcgacccc aaaaaacttg atttgggtga

4021 tggttcacgt agtgggccat cgccctgata gacggttttt cgccctttga cgttggagtc

4081 cacgttcttt aatagtggac tcttgttcca aactggaaca acactcaacc ctatctcggg

4141 ctattctttt gatttataag ggattttgcc gatttcggcc tattggttaa aaaatgagct

4201 gatttaacaa aaatttaacg cgaattttaa caaaatatta acgtttacaa ttttatggtg

4261 cactctcagt acaatctgct ctgatgccgc atagttaagc cagccccgac acccgccaac

4321 acccgctgac gcgccctgac gggcttgtct gctcccggca tccgcttaca gacaagctgt

4381 gaccgtctcc gggagctgca tgtgtcagag gttttcaccg tcatcaccga aacgcgcgag

4441 acgaaagggc ctcgtgatac gcctattttt ataggttaat gtcatgataa taatggtttc

4501 ttagacgtca ggtggcactt ttcggggaaa tgtgcgcgga acccctattt gtttattttt

4561 ctaaatacat tcaaatatgt atccgctcat gagacaataa ccctgataaa tgcttcaata

4621 atattgaaaa aggaagagta tgagtattca acatttccgt gtcgccctta ttcccttttt

4681 tgcggcattt tgccttcctg tttttgctca cccagaaacg ctggtgaaag taaaagatgc

4741 tgaagatcag ttgggtgcac gagtgggtta catcgaactg gatctcaaca gcggtaagat

4801 ccttgagagt tttcgccccg aagaacgttt tccaatgatg agcactttta aagttctgct

4861 atgtggcgcg gtattatccc gtattgacgc cgggcaagag caactcggtc gccgcataca

4921 ctattctcag aatgacttgg ttgagtactc accagtcaca gaaaagcatc ttacggatgg

4981 catgacagta agagaattat gcagtgctgc cataaccatg agtgataaca ctgcggccaa

5041 cttacttctg acaacgatcg gaggaccgaa ggagctaacc gcttttttgc acaacatggg

5101 ggatcatgta actcgccttg atcgttggga accggagctg aatgaagcca taccaaacga

5161 cgagcgtgac accacgatgc ctgtagcaat ggcaacaacg ttgcgcaaac tattaactgg

5221 cgaactactt actctagctt cccggcaaca attaatagac tggatggagg cggataaagt

5281 tgcaggacca cttctgcgct cggcccttcc ggctggctgg tttattgctg ataaatctgg

5341 agccggtgag cgtgggtctc gcggtatcat tgcagcactg gggccagatg gtaagccctc

5401 ccgtatcgta gttatctaca cgacggggag tcaggcaact atggatgaac gaaatagaca

5461 gatcgctgag ataggtgcct cactgattaa gcattggtaa ctgtcagacc aagtttactc

5521 atatatactt tagattgatt taaaacttca tttttaattt aaaaggatct aggtgaagat

5581 cctttttgat aatctcatga ccaaaatccc ttaacgtgag ttttcgttcc actgagcgtc

5641 agaccccgta gaaaagatca aaggatcttc ttgagatcct ttttttctgc gcgtaatctg

5701 ctgcttgcaa acaaaaaaac caccgctacc agcggtggtt tgtttgccgg atcaagagct

5761 accaactctt tttccgaagg taactggctt cagcagagcg cagataccaa atactgtcct

5821 tctagtgtag ccgtagttag gccaccactt caagaactct gtagcaccgc ctacatacct

5881 cgctctgcta atcctgttac cagtggctgc tgccagtggc gataagtcgt gtcttaccgg

5941 gttggactca agacgatagt taccggataa ggcgcagcgg tcgggctgaa cggggggttc

6001 gtgcacacag cccagcttgg agcgaacgac ctacaccgaa ctgagatacc tacagcgtga

6061 gctatgagaa agcgccacgc ttcccgaagg gagaaaggcg gacaggtatc cggtaagcgg

6121 cagggtcgga acaggagagc gcacgaggga gcttccaggg ggaaacgcct ggtatcttta

6181 tagtcctgtc gggtttcgcc acctctgact tgagcgtcga tttttgtgat gctcgtcagg

6241 ggggcggagc ctatggaaaa acgccagcaa cgcggccttt ttacggttcc tggccttttg

6301 ctggcctttt gctcacatgt

//

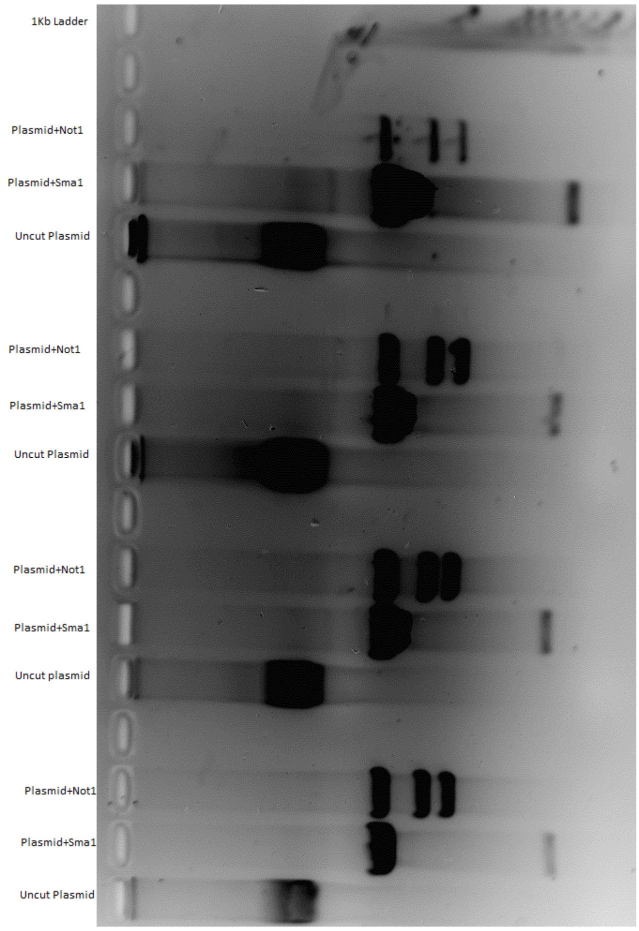

Fig. S.1) Western blot gel presenting the successful insertion of the plasmid.
